# Case of Congenital Obstruction and Imperfect Closure of the Mitral Valve

**Published:** 1854-11

**Authors:** John A. Sweet

**Affiliations:** Physician to the New York Hospital, &c.


Case of Congenital Obstruction and imperfect closure of the Mitral Valve.—By John A. Sweet, M. D., Physician to the New York Hospital, &c. I was called on the 22nd of September, 1851, to visit a young gentleman, twenty-three years of age, in whom I found the physical sign of regurgitation through the mitral orifice of the heart, viz: a bellows murmur with the first sound of the heart, heard over the apex of the organ. The heart did not appear to be much enlarged; the apex seemed to strike in the natural position, and the percussion over the precordial region was quite as clear as is natural. The action of the heart was moderate in degree, but its rhythm was irregular. There was some complaint of palpitation, but much more of dyspnœa. The pulse was very feeble and irregular, and there was slight œdema of the feet, with sigus of effusion into the cavity of the pleura.

The patient was, as I have stated, twenty-three years of age, and a book-keeper. From early childhood, his breathing had been short; but for eight or nine years past this symptom had been more prominent. For the last year it had been still more distressing, and was attended by palpitation. Still, he had been able to follow his occupation until a comparatively recent period. A few days ago, the œdema in his feet commenced. His countenance was pale, but he was not much emaciated; his urine was high-colored and scanty.

Remedies, chiefly of a diuretic character, were administered by the attending physician, Dr. Hobart, but without material benefit. The dropsical tendency increased. Hemoptysis, of a severe form, ensued during the last week of his life, and was frequently repeated. A pulsation was noticed in the epigastrium, as if the right side of the heart was enlarged. Slight jaundice ensued, and delirium followed by coma closed the scene, without a struggle, on the 11th of October.

On *post mortem* examination, twenty-four hours after death, the right cavities of the heart, and the left auricle, were much distended with recent clots of blood; while the left ventricle appeared empty and small, like an appendage to the rest of the organ. The right side was generally enlarged by hypertrophy and dilatation, and the pulmonary artery was larger than the aorta, and nearly as thick. The left auricle was also much enlarged by hypertrophy and dilatation; and besides the soft and recent clots found in it, abundant and old fibrinous clots, partially decomposed, like those we notice in old aneurisms, existed, united to the lining membrane of the auricle by a thin, smooth, and transparent layer of lymph. The same kind of thin layer of lymph adhered to the inner membrane of the pulmonary artery. All the valves and orifices were healthy, except the mitral orifice, which was so contracted as only to admit the end of the forefinger. The structure of the valve was somewhat thickened, but smooth, without ossification, vegetations, or other evidences of inflammation. At each extremity of the valve, there was a seam, or slight depression, each half an inch in length, showing where the opening of the valve should have been continued. The left ventricle was quite natural, except that its walls were rather thinner than usual; some fluid existed in the pericardium.

About two pints of reddish, turbid serum, existed in each pleural sac, with a slight effusion of lymph. The lower lobes of both lungs were not much compressed, but were solidified and of a very dark red color, and giving out on pressure an abundance of dark, viscid blood. No distinct matter of pulmonary apoplexy existed; but both the lower lobes were, I think, passing into this condition.

There was slight cirrhosis of the liver. The other abdominal organs presented nothing abnormal; there was but little venous congestion.—*N. Y. Med. Times.*

